# Determination of Temperature Dependent Growth Parameters in Psychrotrophic Pathogen Bacteria and Tentative Use of Mean Kinetic Temperature for the Microbiological Control of Food

**DOI:** 10.3389/fmicb.2018.03023

**Published:** 2018-12-05

**Authors:** Andrea De Silvestri, Enrico Ferrari, Simone Gozzi, Francesca Marchi, Roberto Foschino

**Affiliations:** ^1^Department of Food, Environmental and Nutritional Sciences, Università degli studi di Milano, Milan, Italy; ^2^Department of Agricultural and Environmental Sciences, Università degli studi di Milano, Milan, Italy; ^3^CAMST S.C.a.r.l.–La Ristorazione Italiana, Villanova di Castenaso, Bologna, Italy

**Keywords:** *Aeromonas hydrophila*, *Listeria monocytogenes*, *Yersinia enterocolitica*, mean kinetic temperature, growth parameters, *E*_a_ values, food safety

## Abstract

Temperature is the main factor to control the microbial growth in perishable foods. The psychrotrophic pathogen bacteria are microorganisms of concern for food products with extended shelf life in chilling conditions. The aims of this work were two. Firstly, to evaluate growth behavior of *Aeromonas hydrophila* DSM-30187, *Listeria monocytogenes* DSM-20600, and *Yersinia enterocolitica* DSM-27689 strains, at different temperatures (4, 7, 10, 15, 25, and 30°C) and starting cell concentrations (10 and 10^6^ CFU/mL), in order to determine the activation energies (*E*_a_) of the relevant lag phases and growth rates. Secondly, to investigate if Mean Kinetic Temperature (MKT) might be applied in recording temperature devices to alert a thermal abuse in a management control system for food safety. As expected, lag phase and growth rate proved to be heavily affected by temperature whereas the inoculum size did not. The *E*_a_ values involved in the duration of latent periods, calculated on the basis of the Arrhenius model, were comparable for *A. hydrophila* and *L. monocytogenes* strains (from 21.3 to 24.4 kcal/mol), while significantly differed for *Y. enterocolitica* (16.6 kcal/mol). The *E*_a_ values of growth rates were similar for *A. hydrophila* and *L. monocytogenes* strains (from 20.9 to 21.1 kcal/mol), while were considerably lower for *Y. enterocolitica* (from 14.2 to 16.7 kcal/mol). The use of MKT is widespread and well-accepted in pharmaceutical field as convenient method for estimating drugs degradation in relation to storage temperature. The *E*_a_ value of the lag phase found for *L. monocytogenes* (23.9 ± 1.2 kcal/mol) was included in the MKT formula. In this work, the air temperature of two chilling rooms was monitored during the normal operating activity in a catering company for a period of 8 months. The MKT profiles were then compared with those of mean temperatures in different conditions (short or prolonged events of thermal abuse) with the purpose to evaluate if it may be applicable to reduce false alarms without lowering the safety level of stored food.

## Introduction

For foods with high level of water activity and a pH value near to neutrality, temperature is the most important environmental factor to delay microbial spoilage, as it directly affects the duration of lag phase and the growth rate (Jay et al., 2005; Adams and Moss, 2008). Since last century, the introduction of industrial and domestic refrigerators dramatically improved the food safety and quality by allowing perishable products to be stored for longer periods, with benefit for the whole food chain, from primary production (harvesting/milking/slaughtering/fishing) to manufacturing, storage, transportation, market distribution and catering services.

Convenience foods (Brunner et al., 2010), including ready-to-eat (RTE) products, ready-to-cook (RTC) and snack foods, have recently met a commercial blast in Hotellerie-Restaurant-Caféterias (HoReCa) supply chain, catering services and at home distribution, supported by their ease-to-use feature, enhanced shelf life and saving time process. Nevertheless, most of these products need an efficient chilling system and temperature maintenance throughout the distributive network to comply food safety standards and retain the sensorial and nutritional properties. In particular, the catering companies provide ready meals in different communities like schools, hospitals, nursing homes for the elderly, corporate canteens (Michino and Otsuki, 2000; Rosset et al., 2004; Foskett and Ceserani, 2007) sometimes located far from the manufacture plants. Foodborne outbreaks due to unfitted behaviors in retail and food services have been reported worldwide (Todd et al., 2008; Todd, 2013; Faour-Klingbeil et al., 2016; Chai et al., 2017); indeed, about 25.5% of European cases were originated in HoReCa segment, while 16.7% in catering services of communities (canteens, kindergartens, schools, hospitals) (EFSA, 2017). Interruption of cold chain during the transport and distribution of food products has been shown to be one of the main risk factors for the development of microorganisms leading to foodborne illness or food deterioration (Jay et al., 2005; Adams and Moss, 2008). Actually, during the shelf-life, the composition of food microbiota is not static, also in controlled chilling conditions, and the amount of time and the temperature at which the food is maintained have a great impact on food safety and quality (Rosset et al., 2004; Heaton and Jones, 2008).

Among the bacterial pathogens, some species are psychrotrophic, namely able to grow at refrigeration temperatures. Within this group, *Listeria monocytogenes* is the most important foodborne pathogen, causing human listeriosis whose lethality has been estimated from 20 to 30% (Buchanan et al., 2004). Due to its ubiquity and stress tolerance to the usual conditions in food processing (Ferreira et al., 2014; NicAogáin and O'Byrne, 2016), *L. monocytogenes* has been identified as target for a mandatory safety criterion (European Commission, 2007). The prevalence of this pathogen has been extensively studied and considered in seafood products (Jami et al., 2014), raw meats (Adley and Dillon, 2011), cheeses (Van Asselt et al., 2017), and RTE foods (Lianou and Sofos, 2007; Currie et al., 2015). A well-known epidemic case occurred in catering activities at primary and secondary schools in northern Italy in 1997 (Aureli et al., 2000) and the scientific literature concerning the persistence of *L. monocytogenes* in this sector have recently reviewed by Osimani and Clementi (2016). The potential contribution of environmental and technological factors in *L. monocytogenes* contamination of RTE foods are under investigation by EFSA Panel on Biological Hazards (EFSA, 2018) to infer the trend of human listeriosis incidence rates throughout a quantitative microbiological risk assessment model.

Because of its increasing antibiotic resistance (Stratev and Odeyemi, 2016) and spreading in aquatic environment and related products (Tuševljak et al., 2012; Hoel et al., 2015), *Aeromonas hydrophila* represents an interesting foodborne pathogen, as causative agent of severe gastroenteritis (Daskalov, 2006). The ability to grow at low temperature and the osmotolerance are determining factors for *A. hydrophila* to become prevalent in sea foods (Papadopoulou et al., 2007; Abd-El-Malek, 2017), salted meat products (Manna et al., 2013; Praveen et al., 2016) and refrigerated minimally processed foods (Xanthopoulos et al., 2010; Nagar et al., 2011). The occurrence and survival of this microorganism in foods have been reviewed by Stratev et al. (2012).

Lastly, *Yersinia enterocolitica* is another current public health concern with a marked psychrotrophic trait (Røssvoll et al., 2014; Ye et al., 2014), causing yersiniosis in humans (Bancerz-Kisiel and Szweda, 2015). It is the third most commonly reported zoonosis in the European Union (EFSA, 2018). It has been isolated from a variety of raw and processed meats, in particular pork (Van Damme et al., 2015; Le Guern et al., 2016) and beef carcasses (Blagojevic and Antic, 2014), raw milk and derived products (Jamali et al., 2015; Özdemir and Arslan, 2015), and RTE salads (Xanthopoulos et al., 2010; Losio et al., 2015; Söderqvist et al., 2016).

Therefore, there is a persistent demand of techniques able to monitor and manage the thermal history of foodstuffs in order to prevent risks for the consumers. Recording devices, like data-loggers, which simply store the temperature/time profile, are commonly applied in the food sector. However, in centralized kitchens and catering distribution services the simple detection of the current temperature during the operation can frequently lead to the occurrence of non-compliances in the moments of higher operating activity. An on-line method to determine and predict the influence of a temperature abuse on the growth of potential pathogen bacteria throughout cold keeping is still strongly required. In pharmaceutical field, the Mean Kinetic Temperature (MKT) parameter is used to easily control the impact of temperature variations on drugs quality, since it shows the overall effect of temperature fluctuations during storage (U. S. Food Drug Administration, 2003; Brown et al., 2006; Seevers et al., 2009). The MKT is defined as a single derived temperature that, if maintained during a defined time, produces the same thermal effect on a biological substance or product as a sequence of lower or higher temperatures would do for an equivalent period of time (Haynes, 1971). It is an index derived from the temperature variations with reference to a specific event, which is critical for the stability of the product. The value of the activation energy (*E*_a_) of a chemical degradation reaction is normally used for determining the impact of the temperature change in the stability testing of pharmaceutical products (Anderson and Scott, 1991). It varies from 5 to 240 kJ/mol, with a mean value of about 83 kJ/mol, that is approximately corresponding to 19.8 kcal/mol (Kommanaboyina and Rhodes, 1999; Seevers et al., 2009). At the same way, the *E*_*a*_ of a growth parameter (e.g., lag phase) might represent a reliable marker in food safety to assess the risk of microbial growth. Studies on the use of MKT in food chains are scarce and it would be worth investigating if an application could be possible to facilitate temperature monitoring in operations with tight working times, such as catering activities. The main purposes of this study have been (i) to investigate the growth kinetics of some psychrotrophic pathogen bacteria at different temperatures in order to get the activation energies of the relevant lag phases and growth rates; (ii) to insert *E*_a_ value of lag phase into the MKT formula; (iii) to verify if the MKT might be used within a suitable alert tool of a management control system, in case of thermal abuse during the maintenance of cool chain.

## Materials and Methods

### Test Microorganisms and Growth Conditions

The psychrotrophic strains used in this work were *A. hydrophila* subsp. *hydrophila* DSM-30187, *L. monocytogenes* DSM-20600 (1/2a serotype) and *Y. enterocolitica* subsp. *enterocolitica* DSM-27689 (serotype 8 biovar 1). Fresh cultures were grown in Tryptic Soy Broth (TSB, Merck, Darmstadt, Germany) at 30°C for 24–48 h. Stock cultures were prepared in TSB and stored at −80°C in the same medium added with glycerol at final concentration of 25% (v/v). Cells were reconstituted by streaking or enumerated by plate count technique on Tryptic Soy Agar plates (TSA, Merck), at 30°C for 24 h in the case of *A. hydrophila* and *Y. enterocolitica*, at 37°C for 48 h for *L. monocytogenes*.

A calibration curve between Optical Density (OD) values at 600 nm and CFU/mL was made for each strain, in order to get the cell concentration to be inoculated into the flasks for the growth experiments. A UV-visible spectrophotometer (Jenway, model 7315, Bibby Scientific Limited, Stone, UK) was used for the optical measurements. A plate count technique was performed to obtain the enumeration of the bacterial cells after decimally diluting in Peptoned Water (Merck), spreading of appropriate aliquots on TSA plates and incubating at the above-mentioned conditions. The growth experiments were carried out in 500 mL polycarbonate Erlenmeyer flasks with DuoCAP® (TriForest, Irvine, USA) containing 100 ml of sterile Brain Heart Infusion broth (BHI, Liofilchem, Roseto degli Abruzzi, Italy) adjusted at pH 6.7, with estimated a_w_ = 0.997. This culture medium has been chosen as a simulant of a real food containing all nutrients for a good growth of the tested microorganisms, in order to set up the worst conditions that can occur. The experimental plan provided for two initial level of inoculum (10 cell/ml and 10^6^ cell/ml), six temperatures of incubation 4, 7, 10, 15, 25, and 30°C ± 0.5°C (Fiocchetti Scientific Refrigerator, Luzzara, Italy and Thermo Scientific Heraeus Incubator, Waltham, U.S.A.), static conditions and three independent replicates for each trial. Depending on the incubation temperature of the test, flasks containing medium were cooled or warmed 24 h before the inoculum. The temperatures were monitored with data-loggers device (LogTag® TRED30-7 ± 0.5°C for −20°C ~ +40°C).

During the incubation period, the cell concentration was monitored at regular time intervals (a) by plate count for experiments starting from an inoculation of about 10 cell/ml and (b) by OD_600nm_ measurement for the experiments starting from an inoculation to about 10^6^ cells /ml, as previously described. Experimental values of cell concentration were fitted with DMFit program (ComBase, US Department of Agriculture, U.S.A.) using the Baranyi and Roberts (1994) model to estimate the lag phase duration and the growth rate. *R*^2^ value was calculated to evaluate the fitting among the experimental points and the predictive model.

### Determination of Activation Energy by Arrhenius Model

The Arrhenius model (Equation 1) was used to describe the temperature dependence on microbial growth. The activation energy was obtained by linear regression using Excel 2016 software (Office 2016, Microsoft Corporation, Redmond, U.S.A.) for both lag phase and growth rate at the two inoculum sizes. The parameters of the Arrhenius equation are:

(1) $$\begin{array}{l} \text{ln }k=\text{ln }A-\;\frac{E_{a}}{RT} \end{array}$$

where:

A is a constant for each chemical reaction that defines the rate due to frequency of collisions; *E*_a_ is the activation energy (kcal/mol); R is the gas constant [1.986 × 10^−3^ (kcal/mol K)]; T is the temperature in Kelvin degree. The factor -*E*_a_/RT is the probability that any given collision will result in a reaction. This equation, derived from thermodynamic laws and developed for reversible chemical reaction gives the temperature dependence for a high number of complex physical, chemical, and biological events (Calligaris et al., 2004). A plot of ln *k* vs. the reciprocal of absolute temperature should give a straight line, whose slope is the activation energy divided by the gas constant (*E*_a_/R).

The analysis of variance (ANOVA) was carried out to investigate the influence of temperature and inoculum size on growth parameters. Data were elaborated by Statgraphics Plus software for Windows (version 4.0, Statistical Graphical Corp., 1999). Tukey HSD test was used to evaluate the difference among the mean values. Plots were done by using IBM SPSS Statistics package (version 25, IBM Corporation, 215).

### Application of the Mean Kinetic Temperature

The MKT (Equation 2) is defined as the isothermal temperature that corresponds to the kinetic effects of a time-temperature distribution and is determined using Haynes' equation (Haynes, 1971), where temperature data obtained at defined intervals are considered. The equation for MKT determination is:

(2) $$\begin{array}{l} {\text{T}}_{\text{MKT}=}\frac{\frac{E_{a}}{R}}{-\ln\left(\frac{e^{\left(\frac{-Ea}{RT_{1}}\right)}+\;e^{\left(\frac{-Ea}{RT_{2}}\right)}+\;\ldots .\;+{e\;}^{\left(\frac{-Ea}{RT_{n}}\right)}}{n}\right)\;} \end{array}$$

where:

*E*_a_ is the activation energy (kcal/mol); R is the gas constant (1.986 10^−3^ kcal/mol K); n is the total number of time periods over which data are collected; temperatures are in Kelvin degree.

The MKT model was applied to the thermal profile of two chiller rooms, respectively, of 30 and 24 m^3^ sizes (DANFOSS, model MTZ 28JE4A) containing meats (the first one) and fruits and RTE vegetables (the second one). The temperatures were monitored using data-loggers device PEGO 200 EXPERT (± 0.5°C, range −45 ~ +45°C). It was then possible to observe the time variability of the temperature with rises and falls, as well as of duration, and the relating effects on a specific reaction, in this case the growth of pathogen microorganisms. The air temperature profiles of two chilling rooms during the normal working activity of an Italian catering company were recorded and analyzed every 15 min for a period of 8 months.

## Results

### Calibration of Microbial Data

The calibration lines obtained by plotting log OD_600nm_ values vs. log plate counts showed high determination coefficients for all the tested strains in the range of OD_600nm_ values 0.1–1.5 corresponding to approximately 10^6^-10^9^ CFU/ml. In particular, the *R*^2^ was 0.95 for *A. hydrophila*, 0.96 for *L. monocytogenes* and 0.98 for *Y. enterocolitica*. A logarithmic transformation was used to stabilize the variance, according to Chorin et al. (1997) and Francois et al. (2003); deviations from linearity were observed for values lower than 10^6^ CFU/ml as the previous authors reported. Augustin et al. (1999) pointed out that the growth rate is underestimated when the cell concentration calculated by OD measurements is lesser than the detection limit; if the case, they suggested dividing the growth rate values by a constant factor. In the present work, no adjustment was needed because the inoculum size (10^6^ cell/ml) was very close to the actual detection limit. Moreover, Baty et al. (2002) demonstrated that optical density measurement and the plate count protocol provide comparable results, if the same model is used to fit the data, as in our study.

Consequently, the cell concentration was monitored by plate count technique for experiments starting from an inoculum size of about 10 cell/ml and by OD_600nm_ measurements for the experiments starting from an inoculum of about 10^6^ cells/ml.

### Determination of Growth Parameters

**Figure 1:**
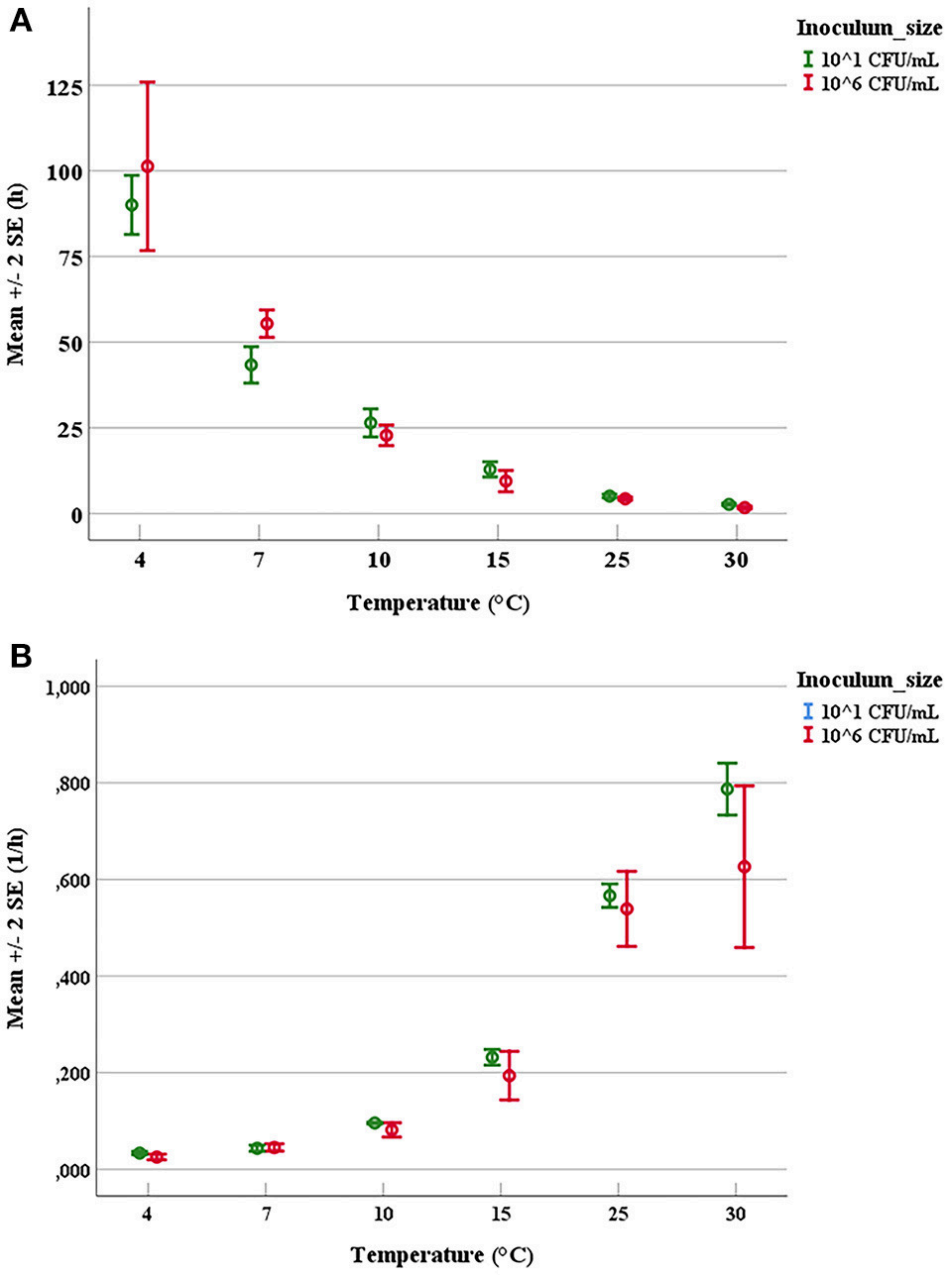
Mean values and relative error bars of lag phases **(A)** and growth rates **(B)** observed at different temperatures and inoculum sizes for *Aeromonas hydrophila* DSM-30187 strain.

**Figure 2:**
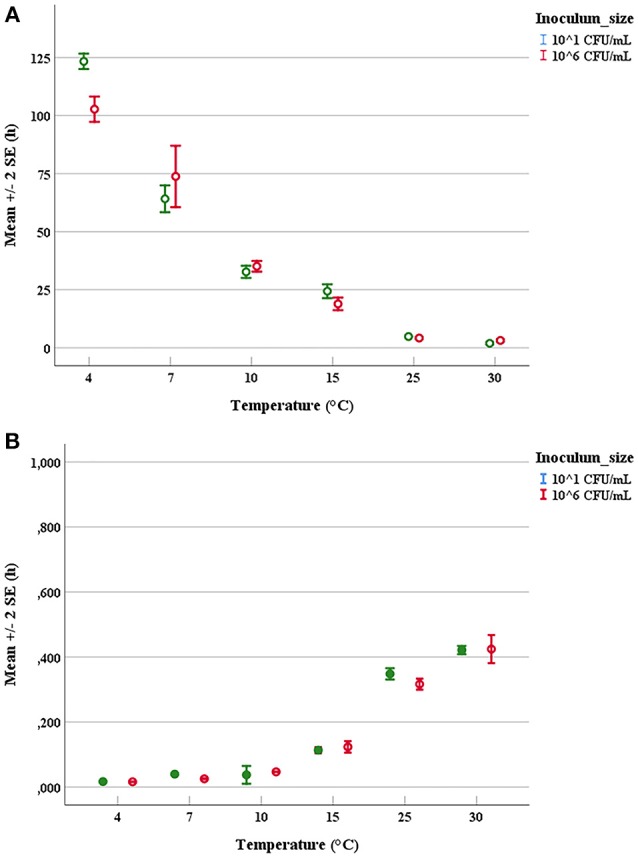
Mean values and relative error bars of lag phases **(A)** and growth rates **(B)** observed at different temperatures and inoculum sizes for *Listeria monocytogenes* DSM-20600 strain.

**Figure 3:**
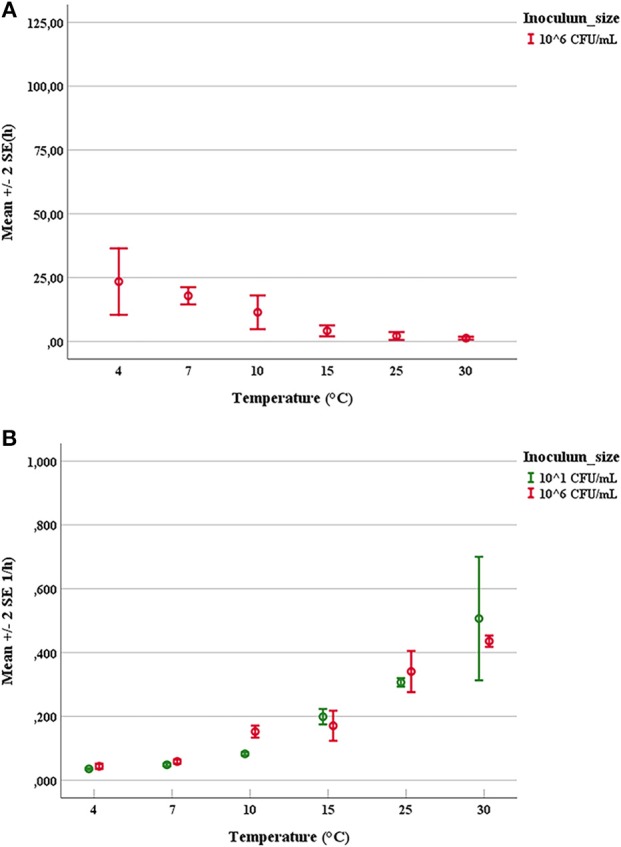
Mean values and relative error bars of lag phase times **(A)** and growth rates **(B)** observed at different temperatures and inoculum sizes for *Yersinia enterocolitica* DSM-27689 strain.

The duration of latent periods and the growth rates were calculated through the growth curves generated from the interpolation of experimental points by using DMFit program of Combase based on the equation of Baranyi and Roberts (1994) (data not shown). Results obtained from the OD_600nm_ measurements were previously converted into cell numbers according to the calibration curve, specific for each strain. The experimental points showed a good fitting (*R*^2^ ≥0.95) with the predictive model for all tested microorganisms. Mean values and relative error bars of lag phase times (Figure 1A) and growth rates (Figure 1B) at different temperatures and inoculum sizes for *A. hydrophila* have been shown. The corresponding data observed in trials with *L. monocytogenes* have been exposed in Figure 2A for lag phase times and in Figure 2B for growth rates, whereas those detected with *Y. enterocolitica* have been reported in Figures 3A,B for lag phase times and growth rates, respectively. In general, growth kinetics of the three psychrotrophic bacteria were similar: the lag phase duration drops as the temperature increases from 4 to 30°C and, as expected, the opposite occurs for the growth rate. Noteworthy, it was not possible to determine the latent period of *Y. enterocolitica* (Figure 3A) for the experiments performed at the lowest inoculum size (10 cell/mL) since a rapid increase in cell counts was always observed, even minimizing sampling times for analysis.

The ANOVA of the obtained data revealed that the starting level of cell concentration did not affect neither the lag phase duration nor the growth rate (Table 1), since significant differences were observed only for *L. monocytogenes* in the case of growth rate values and for *Y. enterocolitica* in the case of lag phase values. As Robinson et al. (2001) already shown, current results confirm that the duration of latent period and the generation time are independent by the inoculum size, if the bacterial growth was carried out in optimal cultural conditions. Conversely, the temperature proved to be a key factor in determining significant difference (*p* < 0.001) between mean values for both the investigated growth parameters. Furthermore, for each strain, the averages of the growth rates did not disclose any significant differences at the lowest temperatures, whereas they became different starting from 15°C (Table 2). Interestingly, the duration of latent periods at 4, 7, 10°C for *Y. enterocolitica* were indistinguishable among them and shorter than those of the other tested strains.

**Table 1:** ANOVA of data and relevant between-subject effects obtained from values of growth parameters for the investigated psychrotrophic pathogens.

**Strain**	**Growth parameter**	**Source**	**DF**	**Sum of squares**	**Mean square**	**F-Ratio**	***(Prob > F) p***
*A. hydrophila* DSM-30187	Lag phase	Model	11	39344.483	3576.77	74.836	<0.001
		Error	24	1147.073	47.79		
		Total	35	40491.556			
		Inoculum size (I.s.)	1	53.047		1.109	0.303
		Temperature (T)	5	38897.545		162.769	<0.001
		I.s. × T	5	393.891		1.648	0.186
	Growth rate	Model	11	2.536	0.230	90.659	<0.001
		Error	24	0.061	0.002		
		Total	35	2.597			
		Inoculum size (I.s.)	1	0.015		5.980	0.022
		Temperature (T)	5	2.494		196.115	<0.001
		I.s. × T	5	0.027		2.139	0.095
*L. monocytogenes* DSM-20600	Lag phase	Model	11	56099.781	5099.98	292.607	<0.001
		Error	24	418.307	17.43		
		Total	35	56518.088			
		Inoculum size (I.s.)	1	45.114		2.588	0.121
		Temperature (T)	5	55267.689		634.187	<0.001
		I.s. × T	5	786.978		9.030	<0.001
	Growth rate	Model	11	0.968	0.088	406.793	<0.001
		Error	24	0.005	0.0002		
		Total	35	0.973			
		Inoculum size (I.s.)	1	0.004		21.154	0.001
		Temperature (T)	5	0.951		878.998	<0.001
		I.s. × T	5	0.012		11.716	<0.001
*Y. enterocolitica* DSM-27689	Lag phase	Model	5	1250.5894	250.118	8.642	0.001
		Error	12	347.3133	28.943		
		Total	17	1597.9028			
		Inoculum size (I.s.)	1	907.01361		62.676	<0.001
		Temperature (T)	5	625.29472		8.642	<0.001
		I.s. x T	5	625.29472		8.642	<0.001
	Growth rate	Model	11	0.876	0.0796	28.010	<0.001
		Error	24	0.068	0.0028		
		Total	35	0.944			
		Inoculum size (I.s.)	1	0.0001		0.045	0.833
		Temperature (T)	5	0.858		60.353	<0.001
		I.s. × T	5	0.018		1.259	0.313

**Table 2:** Mean values of growth parameters at different temperatures calculated for the investigated psychrotrophic pathogens.

**Strain**	**Temperature (°C)**	**Growth parameter**	**Mean value**
*A. hydrophila* DSM-30187	4	Lag phase (h)	95,700^a^
	7		49,383^b^
	10		24,617^c^
	15		11,133^c, d^
	25		4,683^d^
	30		2,167^d^
	4	Growth rate (1/h)	0,029^a^
	7		0,044^a^
	10		0,088^a^
	15		0,213^b^
	25		0,553^c^
	30		0,707^d^
*L. monocytogenes* DSM-20600	4	Lag phase (h)	113,067^a^
	7		68,983^b^
	10		33,900^c^
	15		21,633^d^
	25		4,517^d^
	30		2,550^d^
	4	Growth rate (1/h)	0,016^a^
	7		0,032^a^
	10		0,042^a, b^
	15		0,063^b^
	25		0,332^c^
	30		0,423^d^
*Y. enterocolitica* DSM-27689	4	Lag phase (h)	11,717^a^
	7		8,933^a^
	10		5,700^a, b^
	15		2,067^b^
	25		1,067^b^
	30		0,633^b^
	4	Growth rate (1/h)	0,039^a^
	7		0,053^a^
	10		0,117^a, b^
	15		0,185^b^
	25		0,323^c^
	30		0,471^d^

*Values with different superscripts of lowercase letter in the same column for the each strain and growth parameter are significantly different (p < 0.01)*.

### Calculation of Activation Energies for Lag Phases and Growth Rates

**Figure 4:**
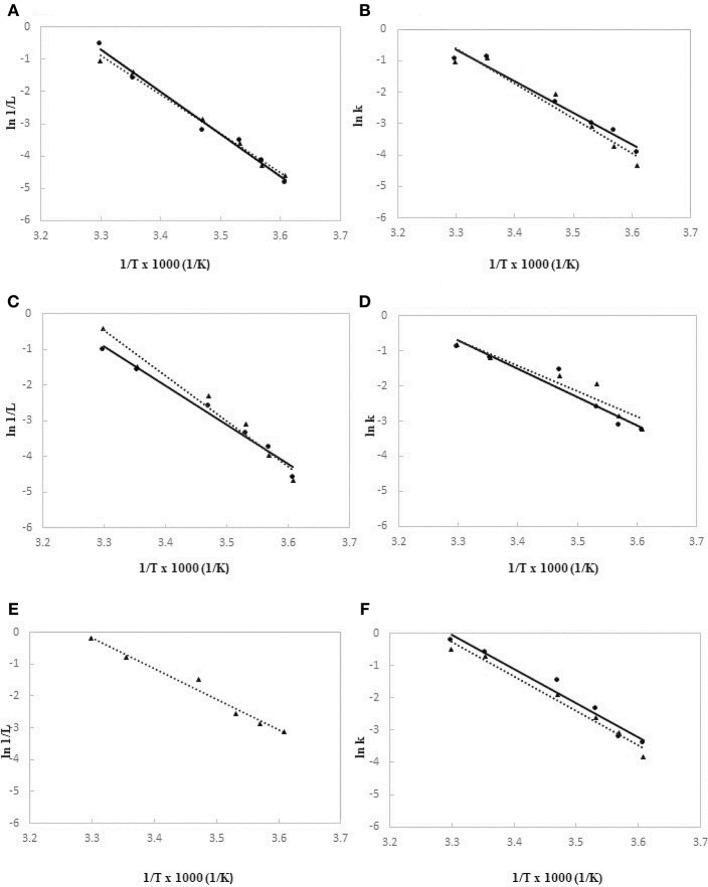
Arrhenius model plots based on lag phase times **(A)** and growth rates **(B)** for *A. hydrophila* DSM-30187, lag phase times **(C)** and growth rates **(D)** for *L. monocytogenes* DSM-20600, lag phase times **(E)** and growth rates **(F)** for *Y. enterocolitica* DSM-27689. Data observed at high level of inoculum (10^6^ CFU/ml) are drawn as ▴ and dotted lines; data at low level of inoculum (10 CFU/ml) are drawn as • and full lines.

The *E*_*a*_ values related to the latent period and growth rate were determined for the investigated strains according to the Arrhenius model, by performing growth tests in a range of temperature from 4 to 30°C. As regards the activation energies of lag phase, the graphs were built by interpolating data of the reciprocal of absolute temperature (1/°K) vs. the reciprocal of natural logarithm of latent period, expressed in hours (ln 1/h), as shown in Figure 4 (Figure 4A, Figure 4C, and Figure 4E for *A. hydrophila, L. monocytogenes* and *Y. enterocolitica*, respectively). At the same way, for the calculation of *E*_*a*_ values of growth rates, the plots were drawn by interpolating data of reciprocal of absolute temperature (1/°K) vs. the natural logarithm of growth rate constant, as presented in Figure 4 (Figure 4B, Figure 4D, and Figure 4F for *A. hydrophila, L. monocytogenes*, and *Y. enterocolitica*, respectively). In the case of *Y. enterocolitica* only the plot regarding the experiments at 10^6^ cell/ml inoculum is reported (Figure 4E), because at 10 cell/ml inoculum the lag phase was not noticed, even at the lowest temperatures.

Table 3 summarized the calculated *E*_*a*_ values based on the observed duration of latent periods and growth rates for each tested strains at two level of inoculum size. *A. hydrophila* and *L. monocytogenes* showed quite similar results for activation energies of lag phase when cultures started at high cell concentration (10^6^ cells/mL), whereas *Y. enterocolitica* exhibited a significant lower value (*p* < 0.01). Similarly, for the growth rates, no significant differences were found between the *E*_*a*_ values of *A. hydrophila* and *L. monocytogenes*, both at high and low inoculum sizes, whereas *Y. enterocolitica* significantly (*p* < 0.01) unveiled the lowest level of activation energy (14.2 kcal/mol).

**Table 3:** Mean values and relative standard deviation of activation energies for the lag phase and growth rate of the investigated psychrotrophic pathogens at different inoculum sizes.

**Strain**	**Lag phase**		**Growth rate**	
**Inoculum size**	***E_a_* (kcal/mol)**	***R*^2^**	***E_a_* (kcal/mol)**	***R*^2^**
*A. hydrophila* DSM-30187				
10 cell/ml	21.3 ± 0.5^a^^1^	0.98	21.1 ± 0.7^a^	0.96
10^6^ cell/ml	23.6 ± 1.4^a, b^	0.97	20.9 ± 0.8^a^	0.98
*L. monocytogenes* DSM-20600				
10 cell/ml	24.4 ± 1.3^b^	0.98	20.9 ± 1.6^a^	0.97
10^6^ cell/ml	23.4 ± 1.1^b^	0.99	21.0 ± 0.4^a^	0.94
*Y. enterocolitica* DSM-27689				
10 cell/ml	ND^2^		16.7 ± 2.6^b^	0.93
10^6^ cell/ml	16.6 ± 1.3^c^	0.98	14.2 ± 1.2^c^	0.91

*R^2^ values refer to the coefficient of determination of experimental data in the Arrhenius' plots*.

1 *Mean values with different superscripts of lowercase letter in the same column are significantly different (p < 0.01)*.

2 *Not Determined*.

Our results were compared with those retrieved in literature, by collecting *E*_*a*_ values from experimental conditions close to our study, and with those predicted in the same environmental conditions by ComBase software (Table 4). The activation energies calculated in the current work for *L. monocytogenes* were similar to those observed by Diez-Gonzalez et al. (2007). They were also comparable to those determined by Rovere et al. (2009) for *L. innocua* species, which showed values ranging from 21.6 to 25.2 kcal/mol for growth rates. As concerns *Y. enterocolitica*, Sutherland and Bayliss (1994) have validated a predictive model of growth in various foods. From the data of generation times at different temperatures published in the just mentioned article, we were able to calculate the activation energies for the growth rate; these values proved to be very similar to those determined in the present work (Table 4). On the other hand, Daughtry et al. (1997) reported the activation energies of lag phase for *Y. enterocolitica* at two temperatures ranges, 2.8–11.7 and 13.3–24.1°C. In this case, the *E*_*a*_ values were different between them and from those we found, probably because of narrower temperature ranges were considered. Iannetti et al. (2017) recently tested the applicability of predictive models on the responses of *L. monocytogenes* and *Y. enterocolitica* strains to dynamic conditions in traditional Italian pork sausage. The *E*_*a*_ values, calculated on the base of the data reported by these authors, were very close to those observed in our work or estimated by Combase program (Table 4). For *A. hydrophila*, this is the first study that experimentally evaluates the activation energies of the growth rate and lag phase by applying the Arrhenius model; detected values were higher than those obtained with the predictive microbiology software. Finally, the *E*_*a*_ values of lag phase determined by the results of Combase program were always lower than those attained by ours, for all the psychrotrophic pathogen bacteria (Table 4).

**Table 4:** Comparison of mean values and relative standard deviation of the activation energies calculated for lag phases and growth rates of the investigated psychrotrophic pathogens in the current study and those found in literature or predicted by Combase program.

**Tested strains**	**References**	***E*_a_ of lag phase** **(kcal/mol)**	***E*_a_ of growth rate** **(kcal/mol)**
*A. hydrophila* DSM-27689	Current work	22.5 ± 1.5	21.0 ± 0.6
*A. hydrophila*	Combase	18.5	18.5
*L. monocytogenes* DSM-20600	Current work	23.9 ± 1.2	21.0 ± 1.0
*L. monocytogenes* H7776	Diez-Gonzalez et al. (2007)	21.4 ± 5.5	18.5 ± 4.9
*L. monocytogenes* mixed strains^a^	Iannetti et al. (2017)		22.9
*L. monocytogenes*	Combase	23.6	21.3
*Y. enterocolitica* DSM-27689	Current work	16.6 ± 1.3	15.5 ± 2.3
*Y. enterocolitica*	Sutherland and Bayliss (1994)		15.5
*Y. enterocolitica*	Daughtry et al. (1997)	14.3–29.6	ND^b^
*Y. enterocolitica* mixed strains^c^	Iannetti et al. (2017)		15.5
*Y. enterocolitica*	Combase	15.6	15.5

a *L. monocytogenes ATCC 7644 and two wild type strains. serotypes 1/2a and 1/2c. isolated from sausage*.

b *Not Determined*.

c *Y. enterocolitica NCTC 10463 and two wild type strains isolated from sausage*.

### Testing of Mean Kinetic Temperature to Actual Food Storage Condition

Current management systems for food safety based on HACCP method consider the temperature monitoring a key element. Temperature data-loggers with triggered alarms are used in order to warn incoming risks due to thermal abuse, to be managed in the NC (non-compliance) procedures. Fake alarms linked to temperature spikes caused by door opening/closing actions of chilling rooms during high operating times, often happens. On the other hand, the simple arithmetic mean temperature value obtained by continuous recording along a daily or weekly period is not sensitive to the discrete changes that occur over time. Monitor the MKT instead of the actual temperature may be convenient to reduce false alarms, by taking advantage of its inherent characteristic to smooth the peaks and to keep count of variations. This choice has been widely made to establish expiration dating and to evaluate thermal excursion during storage and shipment for drug products (Anderson and Scott, 1991; U. S. Food Drug Administration, 2003).

**Figure 5:**
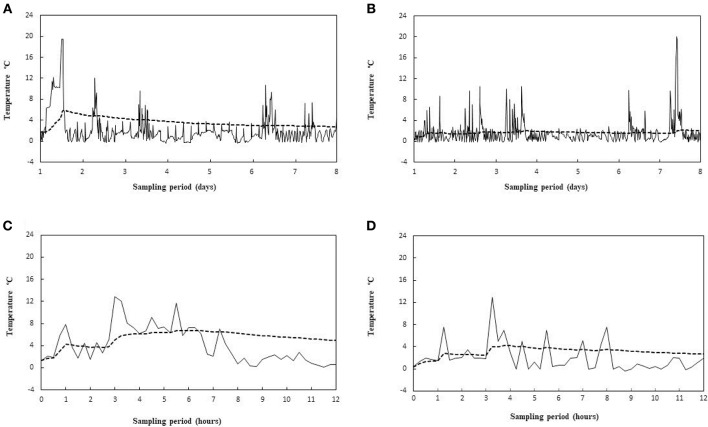
Recorded temperature profiles (—) and relative MKT values ( - - - ) for a 8 days sampling period, showing two different situation of early **(A)** and late **(B)** peak thermal abuses, and for a 12 h sampling period, showing a prolonged thermal abuse **(C)** and a series of short thermal abuses **(D)**.

The MKT temperature was determined from the activation energy of lag phase for each tested strain. The results showed no significant differences in MKT, due to different *E*_a_ values of the three different microorganisms (data not shown), as well as reported by Rovere et al. (2009). Also Seevers et al. (2009) have unveiled that the MKT value is slightly affected by the change in the activation energy levels. Therefore, only the *E*_a_ of the lag phase found for *L. monocytogenes* (average value of *E*_*a*_ = 23.9 ± 1.2 kcal/mol) was included in the MKT formula. In point of fact, this bacterial species is until today the only safety criterion considered in European legislation for food, among the psychrotrophic pathogens. The air temperature of two chilling rooms has been continuously monitored in a catering company for 8 months and the actual thermal profiles have been compared with the relative MKT outlines. The air temperature has been chosen because is more susceptible to thermal changes, while opening the door, than the temperature detected inside foods. While the MKT value depends on the number of observations, some examples of different situations are reported in Figures 5A,B are referred to a 8 days temperature sampling where a major temperature spike occurred in an early stage (Figure 5A) or in a late one (Figure 5B). First and foremost, in both situations the MKT (dotted lines) seems to flatten too much the temperature peaks, but this is due even to the lengthy time interval chosen (see later). Furthermore, the MKT reached a maximum value of 5.7°C in Figure 5A whereas it was only of 2.8°C in Figure 5B. Although more responsive than the arithmetic mean (Table 5), the MKT has a different sensitivity depending on the temporal position of the most pronounced temperature peaks, and this is not eligible for a general usage solution. In order to limit the drawbacks just mentioned, we considered shorter time intervals (12 h). Two other different situations are matched: a pattern of prolonged door opening period is reported in Figure 5C, while an example of a series of fast door opening/closing periods is shown in Figure 5D. It's evident that MKT is less sensitive in the last condition. This is a positive result because in this way it is possible to avoid false alarms caused by incident temperature peaks, remaining relatively sensitive in the situations of prolonged openings shown in Figure 5C. This effect was projected on the temperature data collection by identifying 33 events of prolonged door opening and 42 events of short door opening (Table 5). The duration of the door opening is marked in determining non-compliance condition: the average temperature values of exceeding the threshold limit (4°C) for situations of long permanence are significantly higher than for those of short permanence, obtained both with arithmetic mean and with MKT (Table 5). Remarkable, the MKT is always higher than the arithmetic mean value and even more sensitive since the threshold limit would be exceeded in the observed data. Indeed, in these cases the alarm should have been triggered, proving that the MKT parameter is more cautionary than the simple arithmetic mean. Therefore, choosing a suitable recording time interval, reasonably short, and a consistent threshold of the MKT it would be possible to develop a data-logger suitable for the temperature monitoring in the management systems for daily control of food safety, such as HoReCa or catering services.

**Table 5:** Comparison between arithmetic mean temperature and MKT values observed in two chilling rooms during different periods of door opening frequency along 8 months of monitoring.

	**Chiller 1**			**Chiller 2**		
**Door opening^1^**	**Number of samples^2^**	**Mean T(°C)**	**MKT(°C)**	**Number of samples**	**Mean T (°C)**	**MKT (°C)**
Prolonged	17	3.6 ± 1.5^a A^ ^3^	4.5 ± 1.7^a A^	16	3.1 ± 0.2^a A^	3.9 ± 0.4^a B^
Short	22	2.4 ± 0.6^b A^	2.8 ± 0.7^b B^	20	2.1 ± 0.3^b A^	2.4 ± 0.4^b B^

1 *Prolonged door opening is an event in which four consecutive detections or more upon the 4°C alarm temperature have been recorded; short door opening means that only two consecutive detection upon the 4°C alarm temperature have been occurred*.

2 *Samples are thermal abuse events of prolonged or short door opening*.

3 *For each chiller mean values with different superscripts of lowercase letters in the same column are significantly different; mean values with different superscripts of uppercase letters in the same rows are significantly different (p < 0.01)*.

## Discussion

The maintenance of the cold chain is a specific hygiene measure that is compulsory to assume by European food operators (European Commission, 2004). Among the refrigerated foods, convenience foods have widened the consumers' choices and expectations, nowadays representing the upscale sector in the market. This is also influencing the design and development of products and services in food suppliers and distributers. Due to the extended shelf life in chilling conditions, psychrotrophic pathogen bacteria are the most concerning hazards for these items. In order to be an effective mitigation strategy to manage risks, temperature control systems continuously require to be improved both as personnel training and technical devices to meet the needs of daily practices.

### Determining the Activation Energies of Growth Parameters

Through the application of Arrhenius model, our experimental data fitted well for the calculation of the activation energies, both for lag phases and growth rates. In this way, it was possible firstly describe the *E*_a_ values for *A. hydrophila* and to confirm those already found in literature for *L. monocytogenes* and *Y. enterocolitica*. The tested *Y. enterocolitica* strain showed a different behavior compared to the other two pathogen strains, revealing the ability to grow significantly faster at low temperature. The undetected lag phase duration for *Y. enterocolitica* can be reasonable caused by a smaller *E*_a_ value that allowed an immediate cell multiplication, even at the lowest tested temperature. Furthermore, the use of plate count technique for the enumeration of the microorganisms at low inoculum level (10 CFU/mL) made possible to detect the rapid cell duplication, being this method more sensitive than the optical density measurement by spectrophotometric technique used for the high inoculum level (10^6^ CFU/mL).

According to Diez-Gonzalez et al. (2007) we considered the *E*_a_ of lag phase of pathogen bacteria as a consistent factor relating to the food safety. The basic principle is that the latent period ends when the system reaches that activation energy level for which the microbial cells duplicate. Actually, the rationale for using the refrigeration in food storage is the extension of the lag phase as long as possible or the slowing down of the growth rate of microorganisms by subtracting heat. As far as known, we cannot speculate which are the biochemical reactions underlying the physiological processes involved in the duration of latent period that first accumulate energy to trigger the cell duplication. It has been assumed that lag phase includes hundreds of molecular transformations, such as synthesis of cellular components, repair of molecular damage, transient metal accumulation, all mechanisms adapting to new environmental conditions and preparing for exponential growth (Rolfe et al., 2012). Our results highlight that *A. hydrophila* and *L. monocytogenes* need, on average, a very similar *E*_a_ value of ~21.3–24.4 kcal/mol, whereas *Y. enterocolitica* significantly requires less energy (16.6 kcal/mol) to start cell proliferation. The *E*_*a*_ values found in our work can be useful for future applications to be implemented in devices for the temperature control along the production and distributive food chains.

### Implementation of MKT in Food Control

The frequent non-compliances during high operating times, if alarm systems monitor the actual temperature, or the loss of efficacy in food control, if the mean temperature is calculated after a definite time of recording, brought us to consider the MKT as an alternative or additional parameter in verification procedure for the management of perishable foods. For what having above mentioned, we have included the activation energy of lag phase for *L. monocytogenes* in the MKT formula to investigate its reliability in real situations, thanks to the availability of a huge number of temperature measurements. Due to structure of the equation (2) the duration of the recording time is critical, as the more is the number of observations the less the assumed value by the parameter becomes precautionary in highlighting an overshoot of a threshold limit. Therefore, the application of MKT should be directed to food control in processes with limited time of operation or daily period, such as chilling rooms for HoReCa or catering services with frequent thermal overhangs rather than cold warehouses for long storage. If appropriately designed with reliable algorithm and validated by experimental data through challenge tests, the MKT might be used within a suitable alert tool for the temperature control in order to manage the thermal abuse during the cool chain.

## Author contributions

AD contributed to design the work and revise it, to perform the microbiological testing and elaborate the results. EF contributed to design the work, write, and revise it, to perform statistical analysis and the interpretation of the data. SG contributed to draft and organize the project, to collect temperature data. FM contributed to organize the project, to collect temperature data and revise the work. RF contributed to design, write, and revise the work, to elaborate data and ensure that questions related to the accuracy or integrity of any part of the work were appropriately investigated and resolved.

### Conflict of interest statement

The authors declare that the research was conducted in the absence of any commercial or financial relationships that could be construed as a potential conflict of interest.
